# Phenotypic Heterogeneity and Plasticity of Cancer Cell Migration in a Pancreatic Tumor Three-Dimensional Culture Model

**DOI:** 10.3390/cancers12051305

**Published:** 2020-05-21

**Authors:** Seul-Ki Kim, So Dam Jang, Hyunho Kim, Seok Chung, Jong Kook Park, Hyo-Jeong Kuh

**Affiliations:** 1 Department of Biomedicine & Health Sciences, Graduate School, The Catholic University of Korea, Seoul 06591, Korea; kkssgg-109@hanmail.net (S.-K.K.); ujmmm4@naver.com (S.D.J.); 2 School of Mechanical Engineering, College of Engineering, Korea University, Seoul 02841, Korea; khh8518@korea.ac.kr (H.K.); sidchung@korea.ac.kr (S.C.); 3 Department of Biomedical Science, Research Institute for Bioscience & Biotechnology, Hallym University, Chuncheon, Gangwon-do 24252, Korea; jkp555@hallym.ac.kr; 4 Cancer Evolution Research Center, College of Medicine, The Catholic University of Korea, Seoul 06591, Korea; 5 Department of Medical Life Sciences, College of Medicine, The Catholic University of Korea, Seoul 06591, Korea

**Keywords:** pancreatic cancer, tumor spheroids, 3D co-culture, migration, ECM remodeling

## Abstract

Invasive cancer cell migration is a key feature of metastatic human pancreatic ductal adenocarcinoma (PDAC), yet the underlying mechanisms remain poorly understood. Here, we investigated modes of cancer cell invasion using two pancreatic cancer cell lines with differential epithelial–mesenchymal status, PANC-1 and BxPC-3, under 3D culture conditions. Multicellular tumor spheroids (TSs) were grown in a collagen matrix co-cultured with pancreatic stellate cells (PSCs) using microchannel chips. PANC-1 cells showed individual migration from TSs via invadopodium formation. BxPC-3 cells showed plasticity between collective and individual migration in either mesenchymal mode, with filopodium-like protrusions, or blebby amoeboid mode. These two cell lines showed significantly different patterns of extracellular matrix (ECM) remodeling, with MMP-dependent degradation in a limited area of ECM around invadopodia for PANC-1 cells, or MMP-independent extensive deformation of ECM for BxPC-3 cells. Cancer cell migration out of the collagen channel significantly increased by PSCs and directional cancer cell migration was mediated by fibronectin deposited by PSCs. Our results highlight the phenotypic heterogeneity and plasticity of PDAC cell migration and ECM remodeling under 3D culture conditions. This 3D co-culture model of pancreatic cancer cells and PSCs offers a useful tool for studying cancer cell migration and ECM remodeling to identify and develop potential molecular targets and anti-cancer agents against human PDAC.

## 1. Introduction

Pancreatic ductal adenocarcinoma (PDAC) has one of the worst prognoses of all human cancers, with a 5 year survival rate of less than 6% [1]. This poor prognosis is associated with the aggressive nature of PDAC, characterized by early invasion and metastasis, as well as chemoresistance [2]. Moreover, the highly desmoplastic nature of PDAC, which is related to the underlying mechanisms of cancer progression, further contributes to the poor prognosis [3]. Although anti-desmoplastic therapies have been developed and tested, the results have not been promising to date [4].

Cell migration is a complex and multistep process, which can be classified into different modes based on cell morphology and related mechanisms [5]. Cancer cells move either individually (amoeboid or mesenchymal) or collectively as clusters and form membrane protrusions from dramatic reorganization of the actin cytoskeleton, including lamellipodia, filopodia, and invadopodia [6,7]. When cancer cells invade the surrounding matrix, cells can remodel the matrix to create a tumor-permissive environment [8]. Matrix metalloproteinases (MMPs) are well-known factors involved in priming of the extracellular matrix (ECM) via pericellular proteolysis, which allows the cells to realign matrix fibers to pave a pathway to migration [9]. This reciprocal interaction between migrating cells and the surrounding ECM has emerged as an important mechanism for controlling the migration mode and ECM remodeling, which ultimately determines the invasion potential and progression to metastasis [10]. Therefore, gaining a clearer understanding of the processes and factors involved in mechanotransduction during invasion and migration could offer new targets and strategies for treatments of highly invasive cancers such as PDAC.

The tumor microenvironment, which consists of stromal cells such as cancer-associated fibroblasts, immune cells, and ECM, as well as many soluble factors, provides biochemical and biophysical signals to cells that facilitate tumor progression and invasion [11,12]. Activated pancreatic stellate cells (PSCs) can be transformed into myofibroblast-like cells such as cancer-associated fibroblasts, and hence have a strong impact on the malignant behavior of PDAC [13,14]. Activated PSCs are considered to secrete factors promoting the epithelial-mesenchymal transition (EMT) and act as major contributors of the ECM deposition that constitute the desmoplasia of PDAC [15]. The biochemical and mechanical cues of the ECM may influence metastatic progression [16].

Accumulating evidence indicates that three-dimensionality is not only critical in cell–cell interactions, but also in cell–matrix interactions, which are important for the regulation of cancer invasion [17,18,19]. Recent technical progress has now made it possible to establish 3D cancer cell cultures in conditions that mimic the in vivo tumor microenvironment [20,21]. Using our previously developed microchannel (microfluidic) system recapitulating the in vivo tumor microenvironment [22,23], we aimed to investigate the modes of cancer cell invasion and ECM remodeling using multicellular tumor spheroids (TSs) of two PDAC cell lines, representing a mesenchymal and epithelial cell type, cultured with PSCs in a 3D ECM environment.

## 2. Results

### 2.1. Formation and Growth of Pancreatic TSs in a Collagen Matrix

**Figure 1:**
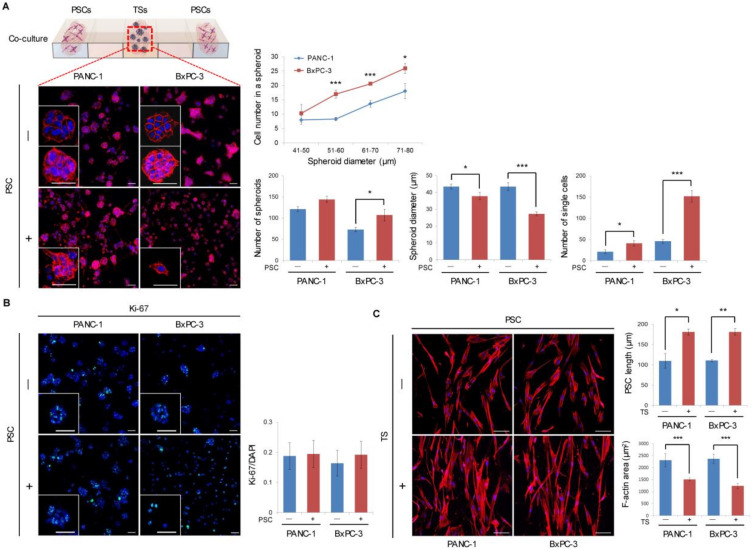
Formation and growth of pancreatic tumor spheroids (TSs) in a collagen matrix with or without pancreatic stellate cells (PSCs). (**A**) Compactness, number, and size of TSs and single-cell dissemination for PANC-1 and BxPC-3 cells in the presence or absence of PSCs. Scale bar: 50 µm. (**B**) Level of the Ki-67 proliferation marker under PSC co-culture conditions. Scale bar: 50 µm. (**C**) Changes in morphology of PSCs when co-cultured with PANC-1 or BxPC-3 TSs. Scale bar: 100 µm. Cells were grown for 5 days in collagen-supported microchannel chips and stained with F-actin, Ki-67, and DAPI. Cells up to 20 μm in diameter were counted as single cells. Three fields covering 80% of the effective area in each channel were imaged per experiment and subjected to analysis. Data were obtained from three separate independent experiments. * *p* < 0.05, ** *p* < 0.01, *** *p* < 0.005.

After 5 days of culture in a collagen matrix, PANC-1 and BxPC-3 cells formed spheroids with an average diameter of 40 µm (range 21–100 µm). BxPC-3 cells showed greater spheroid compaction, aggregating 2-fold more cells compared to PANC-1 cells (Figure 1A). PANC-1 culture showed 1.6-fold more TSs, whereas fewer TSs with more single cells were found to be scattered in BxPC-3 culture. Under PSC co-culture, the number of spheroids increased while the spheroid diameter decreased and the number of single scattered cells significantly increased in both PANC-1 and BxPC-3 cells. There was no difference in the proliferation of cancer cells under PSC co-culture for either cell line, as indicated by staining for the proliferation marker Ki-67 (Figure 1B). Under co-culture in both cell lines, PSCs showed a significant increase in cell length and significant decrease in cell size (F-actin area) with an elongated spindle shape (Figure 1C). It was noteworthy, however, that no difference was observed in the level of activation marker α-SMA (Figure S1A). The number of PSCs migrating out of the collagen channel appeared to increase, but did not reach the level of statistical significance Figure S1B). These changes in tumor cells and PSCs under co-culture clearly indicated a mutual interaction between these cells in the collagen-supported microchannel, although PSCs showed a partial activation.

### 2.2. Expression of EMT-Related Factors in TSs

**Figure 2:**
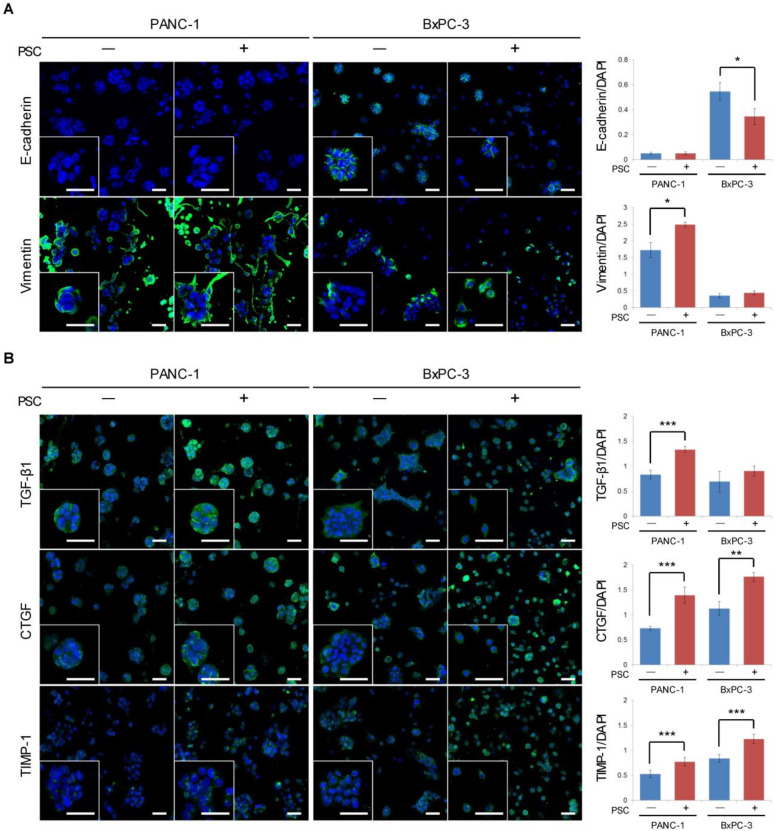
Expression of epithelial-mesenchymal transition (EMT)-related proteins in tumor spheroids (TSs) under PSC co-culture conditions. (**A**) Expression of EMT marker proteins E-cadherin and vimentin. (**B**) Expression of EMT-inducing factors TGF-β1, CTGF, and TIMP-1. Protein expression levels were normalized by nuclear staining with DAPI. Optical sections were acquired at 6 µm (10×) or 2 µm (40×) intervals and stacked into a z-projection. Cells were grown for 5 days in collagen-supported microchannel chips. Three fields covering 80% of the effective area in each channel were imaged per experiment and subjected to analysis. Data were obtained from three separate independent experiments. Scale bar: 50 µm (**A**, **B**). * *p* < 0.05, ** *p* < 0.01, *** *p* < 0.005.

The expression levels of E-cadherin and vimentin were consistent with the EMT traits of PANC-1 and BxPC-3 cells, which are mesenchymal and epithelial type cells, respectively (Figure 2A). Under co-culture conditions, PANC-1 cells showed increased vimentin expression with a concurrent increase in EMT-inducing factors, including TGF-β1, CTGF, and TIMP-1 (Figure 2B). By contrast, BxPC-3 cells showed a decreased level of E-cadherin expression, with no change in vimentin expression (Figure 2A). In BxPC-3 cells co-cultured with PSCs, increased expression levels of CTGF and TIMP-1 were observed, with no change in TGF-β1 expression (Figure 2B).

### 2.3. Differential Modes of Cancer Cell Migration and Focal Adhesion

**Figure 3:**
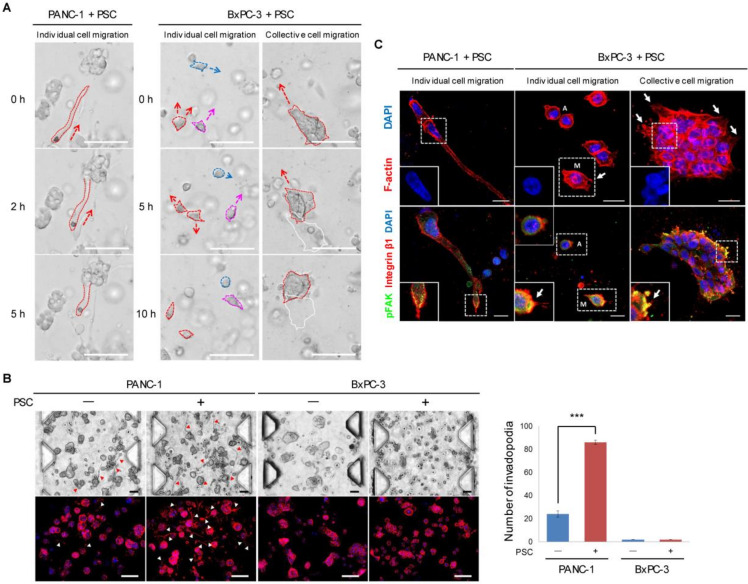
Difference in migration mode and FAK activation between PANC-1 and BxPC-3 cells in a collagen matrix. (**A**) Time-lapse images of cell migration under PSC co-culture conditions, showing differential migration modes. Direction of cell migration is indicated by arrows. Scale bar: 100 µm. (**B**) Increased number of invadopodia (arrowhead) in PANC-1 TSs under PSC co-culture conditions. Scale bar: 100 µm. (**C**) Comparison of podium morphology and expression of pFAK at the leading edge of the protrusion between PANC-1 and BxPC-3 cells under co-culture conditions. A: amoeboid mode. M: mesenchymal mode. White arrow: spike-like filopodia. Scale bar: 20 µm. Three fields covering 80% of the effective area in each channel were imaged per experiment and subjected to analysis. Data were obtained from three separate independent experiments. *** *p* < 0.005.

PANC-1 and BxPC-3 cells showed different modes of migration through the 3D collagen matrix. PANC-1 cells showed an individual cell migration pattern using invadopodia and expressed MT1-MMP [24] (Figure S2), whereas BxPC-3 cells showed individual as well as collective cell migration without invadopodia (Figure 3A). A substantial fraction of PANC-1 cells showed actin-rich protrusions in the form of invadopodia, and the number of these protrusions increased under PSC co-culture conditions (Figure 3B). PANC-1 cells migrating via invadopodia demonstrated deformation of the nuclear shape to the axis of extending invadopodia, and pFAK expression was observed at the limited area of the protrusion front (Figure 3C). BxPC-3 cells demonstrated two different modes of individual cell migration: a mesenchymal mode with spike-like filopodia and an amoeboid mode without protrusion. Collective migration of BxPC-3 TSs appeared as aggregated cells migrating with spike-like filopodia. In BxPC-3 cells migrating in mesenchymal and collective mode, but not those exhibiting amoeboid migration, activation of focal adhesion kinase was observed with dense expression of integrin β1 (Figure 3C). Other pancreatic cancer cell lines with mesenchymal (MIA PaCa-2) and epithelial (Capan-1) characteristics did not show either comparable migration ability or single-cell dissemination in the 3D collagen matrix (Figure S3) and were not considered suitable for the present study.

### 2.4. Remodeling of the Collagen and Fibronectin Matrix

**Figure 4:**
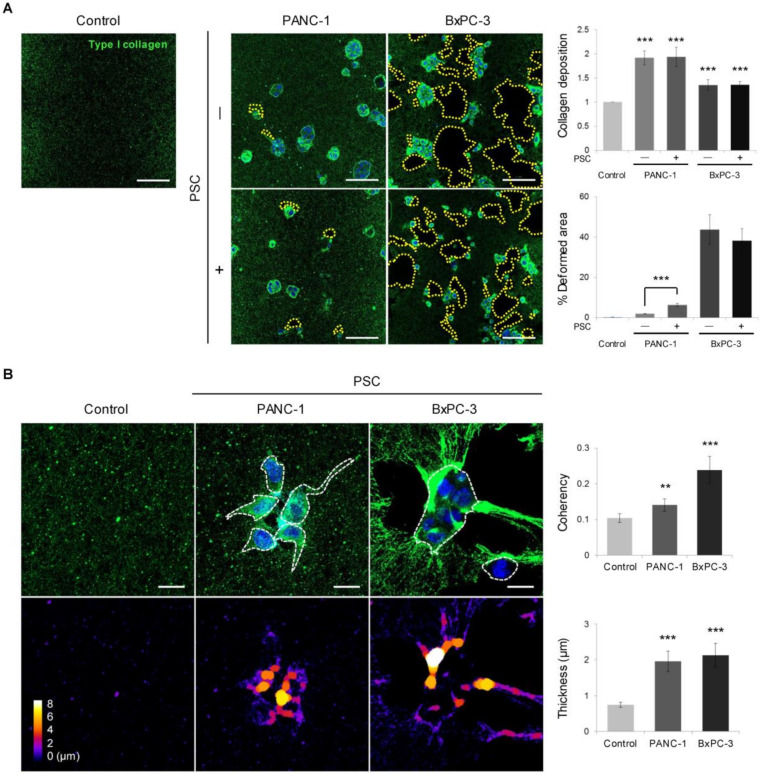
Differential remodeling of the collagen matrix between PANC-1 and BxPC-3 cells. (**A**) Deposition and spatial distribution of PANC-1 and BxPC-3 cells with or without PSC co-culture. Scale bar: 100 µm. (**B**) Changes in collagen structure and organization with respect to coherence and thickness. Scale bar: 20 µm. Control: cell-free matrix. Three fields were imaged per experiments and more than 10 regions of interest were selected for the experiment and subjected to analysis. Data were obtained from three separate independent experiments. ** *p* < 0.01, *** *p* < 0.005.

The deposition of type I collagen significantly increased in the presence of PANC-1 and BxPC-3 cells regardless of co-culture with PSCs (Figure 4A). Deformation of the collagen matrix was observed in less than 10% of the total ECM area in PANC-1 culture, whereas up to 40% of the areas near spheroids comprised void patches in BxPC-3 culture. Under PSC co-culture, the level of collagen degradation increased in PANC-1 cells but not in BxPC-3 cells. Significant changes in collagen fiber orientation and thickness were observed around TSs in the sites of collagen degradation (Figure 4B).

**Figure 5:**
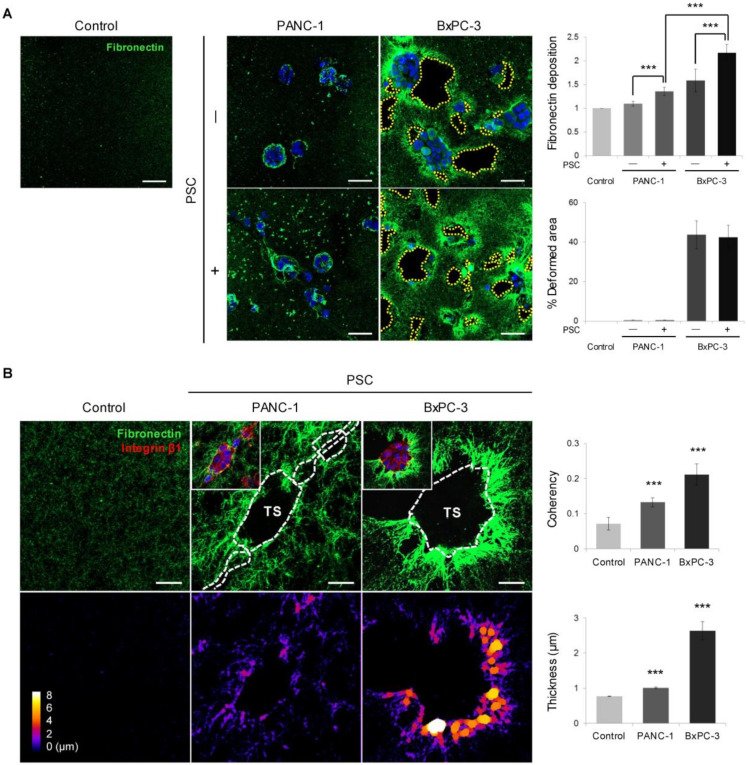
Differential remodeling of the fibronectin matrix between PANC-1 and BxPC-3 cells. (**A**) Effects of PSC co-culture on the expression of fibronectin in TSs. Scale bar: 50 µm. (**B**) Changes in fibronectin structure and organization with respect to coherence and thickness. Scale bar: 20 µm. Control: cell-free matrix. TS: tumor spheroid. Three fields were imaged per experiments and more than 10 regions of interest were selected for experiment and subjected to analysis. Data were obtained from three separate independent experiments. *** *p* < 0.005.

Similar changes were observed in the fibronectin matrix, which is a major factor facilitating cancer cell invasion and migration. PANC-1 cells showed a minimal area of fibronectin deposition, whereas BxPC-3 cells showed extensive distribution around TSs except in the void area of collagen distribution (Figure 5A,B). Fibronectin deposition by BxPC-3 cells increased under PSC co-culture, suggesting that PSCs stimulated fibronectin secretion in the cells via a soluble-factor-mediated interaction. Significant changes in fibronectin fiber orientation and thickness were also observed around TSs (Figure 5B), similarly to the collagen matrix (Figure 4). The low level of fibronectin in the cell-free collagen matrix was attributed to the fibronectin present in FBS-containing media (Figure S4).

### 2.5. Expression of MMPs and Cleaved Collagen

**Figure 6:**
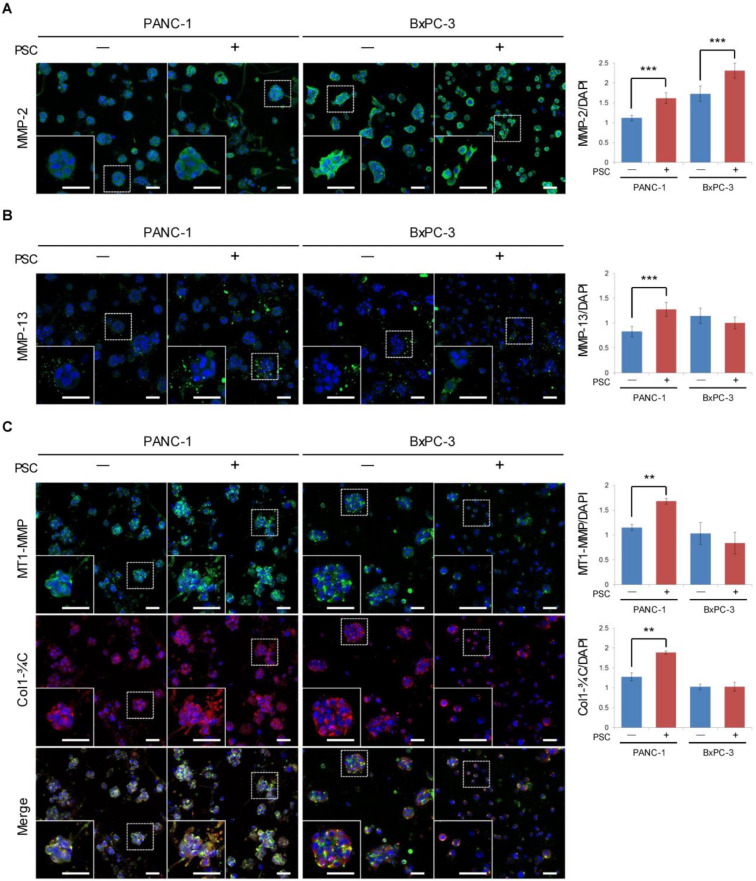
Differential expression levels of matrix metalloproteinases (MMPs) and cleaved collagen in tumor spheroids (TSs) of PANC-1 and BxPC-3 cells. The expression levels of MMP-2 (**A**), MMP-13 (**B**), MT1-MMP, and Col1-¾C (**C**) were normalized by nuclear staining with DAPI. Optical sections were acquired at 6 µm (10×) or 2 µm (40×) intervals and stacked into a z-projection. Three fields covering 80% of the effective area in each channel were imaged per experiment and subjected to analysis. Data were obtained from three separate independent experiments. Scale bar: 50 µm (**A**–**C**). ** *p* < 0.01, *** *p* < 0.005.

To identify the factors determining the differential degree of ECM remodeling, the expression of MMPs was compared between PANC-1 and BxPC-3 cells. Although MMP-2 levels were higher in BxPC-3 cells compared to PANC-1 cells, there was no proportional change in the expression levels of other MMPs such as MMP-13 and MT1-MMP or cleaved collagen (Col1-¾C) with respect to the extensive degree of ECM remodeling detected in BxPC-3 cells (Figure 6). Moreover, cleaved collagen was detected at the peri-spheroid areas but not close to the ECM void area (Figure 6C). In PANC-1 TSs, the level of cleaved collagen increased with concurrent changes in the levels of MMPs such as MMP-2, MMP-13, and MT1-MMP under co-culture conditions, indicating that ECM remodeling in PANC-1 cells may be attributed to MMP-dependent mechanisms. However, BxPC-3 TSs showed a similar level of cleaved collagen expression to that of PANC-1 cells, and no further changes were observed in cleaved collagen, indicating that ECM remodeling in BxPC-3 culture is related to mechanisms independent of major collagen degradation.

### 2.6. Migration of Cancer Cells out of the Collagen Matrix and Contact with PSCs

**Figure 7:**
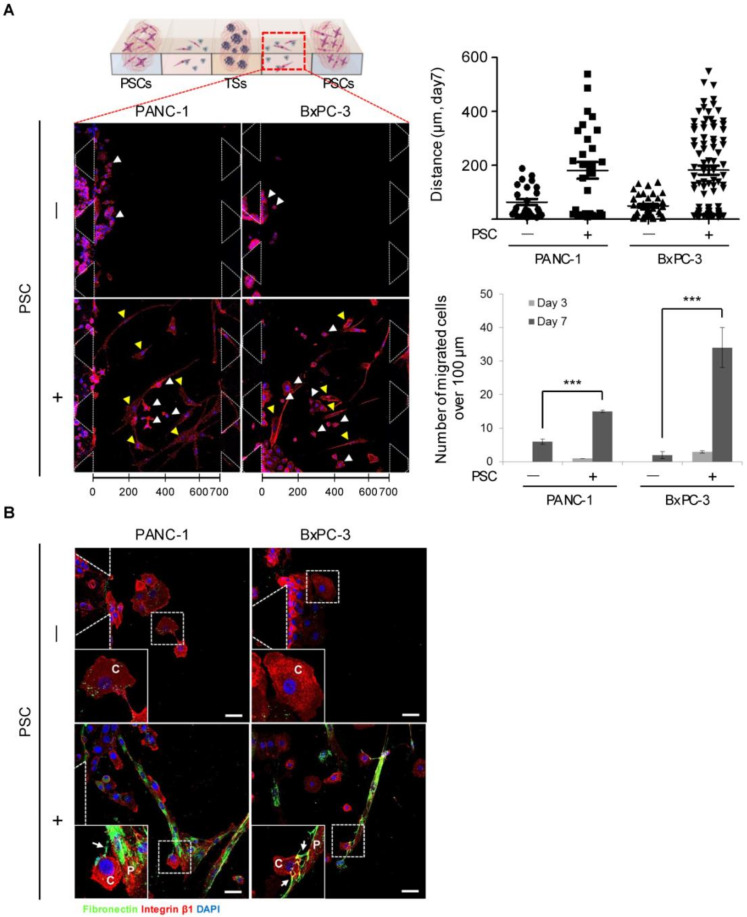
Fibronectin-mediated interaction between cancer cells and PSCs migrating out of the collagen matrix channel. (**A**) Distance and number of migrating PANC-1 or BxPC-3 cells under PSC co-culture conditions. Cells that migrated beyond 100 µm from the respective channels were included in the analysis. (**B**) Cancer cells adhering to fibronectin fibers of spindle-shape PSCs. Scale bar: 20 µm. White arrowhead or C: cancer cells. Yellow arrowhead or P: PSCs. Arrows indicate fibronectin fibers secreted by PSCs. Three fields covering 80% of the effective area in each channel were imaged per experiment and subjected to analysis. Data were obtained from three separate independent experiments. *** *p* < 0.001.

The number of cells migrating out of the collagen channel significantly increased over time and under PSC co-culture in both PANC-1 and BxPC-3 cells (Figure 7A). The cancer cells alone did not show notable migration, whereas approximately 50% of the cells migrated a distance over 200 µm under co-culture conditions. Cancer cells and PSCs were found to make contact with each other in the medium channels following 7 days of co-culture (Figure 7B). PSCs in the medium channels showed fibronectin deposition on the cell surfaces to which the cancer cells attached.

## 3. Discussion

Cancer cell migration within a 3D matrix recapitulates the cellular motility occurring in an interstitial matrix in vivo [25] as a complex 3D structure organized by fibrillar collagens harboring pores of varying sizes can affect the paths of migrating cells [26]. Cell migration on a 2D substrate is quite different from that occurring in the 3D microenvironment of an interstitial matrix. Cells within a 3D ECM typically show a high degree of plasticity, able to adapt their migration modes according to changes in intrinsic and extrinsic factors [17,27]. Therefore, multicellular TSs grown in a 3D matrix may most closely mimic in vivo conditions for the purpose of exploring invasion mechanisms [28]. Accordingly, we used a 3D ECM model system based on a microchannel (microfluidic) chip to investigate the modes and mechanisms of migration in pancreatic cancer cells differing in EMT properties.

PANC-1 and BxPC-3 cells are well-established pancreatic cancer cell lines and are widely used for research [29,30]. However, data on the invasiveness or metastatic potential of these cells in animal models are not yet available [31,32]. The PANC-1 cell line represents a mesenchymal cell type with low E-cadherin and high vimentin expression, whereas BxPC-3 cells are considered to represent an epithelial type with opposite patterns of the expression of these markers [33] (Figure 2A). Under PSC co-culture, PANC-1 and BxPC-3 cells showed further transition towards a mesenchymal phenotype, as demonstrated by increased vimentin and decreased E-cadherin expression, respectively. Although the change was attributed to increased level of EMT-inducing factors such as TGF-β1, CTGF, and TIMP-1 (Figure 2B) in PANC-1 cells, the lack of change in TGF-β1 expression in BxPC-3 cells (Figure 2B) might have been due to *SMAD4* mutation uncoupling intracellular TGF-β autocrine signaling [34]. Spheroid compaction in the two cell lines was consistent with their differences in E-cadherin expression levels, in which BxPC-3 TSs contained about 2-fold more aggregated cells than PANC-1 TSs (Figure 1A). In spite of the epithelial nature and compactness of BxPC-3 spheroids, they showed greater ability for single-cell dissemination than PANC-1 spheroids, which further increased under PSC co-culture. Similarly to the differences in single-cell dissemination within the ECM, migration out of the matrix channel under PSC co-culture conditions was higher in BxPC-3 cells than in PANC-1 cells (Figure 7A). Collectively, these data suggest that E-cadherin-positive epithelial cells do not necessarily confer a lower potential of cell migration in a 3D environment [35]. Nonetheless, loss of E-cadherin expression in disseminated single cells or population heterogeneity in E-cadherin expression cannot be ruled out [36,37], and warrant further investigation.

**Figure 8:**
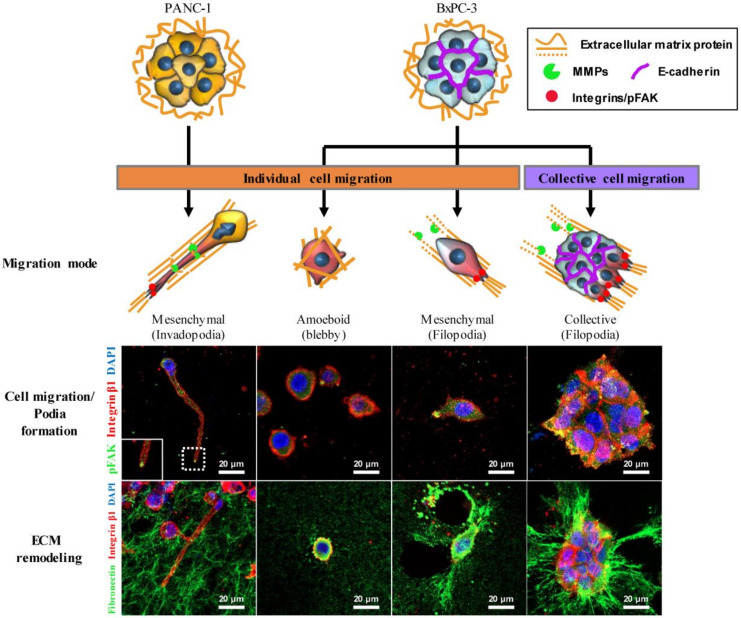
Plasticity and mechanoreciprocity in pancreatic cancer cell migration and cell–ECM interactions. Cancer cells showed individual or collective cell migration in 3D ECM environments. Individual PANC-1 cells formed actin-rich protrusions of the plasma membrane (invadopodia). PANC-1 cells showed organized ECM fibers along the direction of invadopodium growth. Individual BxPC-3 cells appeared as either a rounded shape (amoeboid) without podium formation or as a mesenchymal shape with actin-spike protrusions (filopodia). The mechanoreciprocity of cell–ECM interactions appeared to be associated with integrin-based adhesion. BxPC-3 cells showed extensive ECM deformation and unfolding around the filopodia along with a FAK-mediated traction force. Scale bar: 20 µm.

Cancer cells adapt multiple migration modes depending on cell–cell adhesion and cell–matrix mechanotransduction [38]. Differential modes of cell migration were observed between PANC-1 and BxPC-3 cells in the 3D ECM, and are summarized with corresponding ECM remodeling of fibronectin matrix in Figure 8. PANC-1 cells showed individual cell migration using invadopodia, which was enhanced in parallel with greater invadopodium formation under PSC co-culture (Figure 3B), whereas BxPC-3 cells showed heterotypic migration, including collective and individual cell migration in either mesenchymal or amoeboid mode (Figure 3C). In BxPC-3 cells, actin-spike protrusions in the form of filopodia led the cells in collective and mesenchymal migration, and mechanotransduction occurred as indicated by expression of integrin β1 and pFAK [39,40]. However, no filopodium formation and lack of pFAK expression was observed in BxPC-3 cells showing the amoeboid migration mode (Figure 3C). The amoeboid mode of migration is characterized by round cell morphology without cell–ECM adhesion, and the ability of matrix deformation without proteolysis [41].

Interactions of cells with the ECM are critical for promoting invasion [42,43,44]. The mechanical properties of the ECM are sensed by cells via integrin mechanosensing, leading to activation of focal adhesion protein modules to induce mechanosignaling [45]. This mechanotransduction in turn generates various cellular responses, inducing membrane protrusion and migration [46]. Bi-directional crosstalk occurs, termed mechanoreciprocity, wherein cells not only sense and adjust their behavior to the microenvironment but also modify the ECM with quantitative and qualitative changes [38]. ECM remodeling by migrating cells occurs through both proteolytic degradation and deposition. ECM degradation is executed by cell-derived proteases, including soluble and membrane-anchored MMPs along with the serine protease urokinase plasminogen activator [47,48]. Soluble MMPs not only generate a physical space along the cell–tissue interface but also disrupt wider regions to enable the migration of other cells independent of their proteolytic ability [49]. In PANC-1 TSs co-cultured with PSCs, increased invadopodium formation was correlated with increased levels of MMP expression and cleaved collagen (Figure 6). Invadopodium formation has also been observed in vivo, and recent studies demonstrated direct molecular links between the assembly of invadopodia and metastasis in both mouse models and in human patients [50,51]. In particular, MT1-MMP is enriched at the invadopodia, which degrades ECM proteins and promotes cell invasion [52,53]. In line with these previous findings, we found an overlapping distribution of cleaved collagen and MT1-MMP expression (Figure 6C).

The extensive ECM remodeling observed in BxPC-3 TS cultures seemed to be independent of proteolysis (Figure 4 and Figure 5). The pulling and unfolding of entire ECM layers induced by moving cells are mechanical and reversible events. Several recent studies have suggested that ECM remodeling into parallel fibrils occurs via a mechanical force exerted by migrating cells in collective movement [54]. Collective cell invasion was found to be facilitated on the ECM aligned by an intracellular signaling (FAK)-mediated tensile force in a 3D environment [55]. The directionality of aligned matrix fibrils is considered to provide an efficient path for migrating cells, especially in collective migration [56]. Contractile-force-mediated collagen remodeling has been observed at the tumor–stroma interface to promote cancer cell invasion [57], and long-range stress stiffening of the collagen matrix by highly contractile cancer cells has been reported [58].

The mode of cancer cell migration is commonly classified as either individual (amoeboid or mesenchymal) or collective migration based on the morphology of migration pattern [49]. Collective migration drives the multicellular invasion of many epithelial cancer cells into the peritumor stroma while retaining their cell–cell adhesion, mainly mediated by E-cadherin [59]. Mesenchymal cells, due to their lack of extensive cell–cell adhesion, are considered to migrate in individual modes [60]. One additional pattern of cell migration presents multicellular streaming, characterized by loosely attached or non-adherent individual cells that display amoeboid-like or mesenchymal phenotypes and migrate along the same path [5,6]. Single cancer cells can invade surrounding tissue via protease-dependent mesenchymal migration or protease-independent amoeboid migration, or a combination of both [61,62]. In relation to the relationship between EMT and invasive phenotypes, EMT action as a binary process between fully epithelial and mesenchymal states has been widely accepted. Recent evidence indicates, however, that EMT induces a broad range of intermediate epithelial and mesenchymal stages such as partial EMT states, and that it contributes to phenotypic plasticity of cancer cells in invasion and metastasis, especially in collective cell migration [63,64]. In the present study, BxPC-3 cells showed a combination of collective as well as individual migration in mesenchymal or amoeboid modes (Figure 8), in which the phenotypic heterogeneity and plasticity was evident; this line may therefore serve as a useful model to study the role of partial EMT and underlying mechanisms.

The recent progress in human organs-on-chips technology has allowed reproduction of cancer metastasis in vitro. Although there are challenges that must be overcome, these models may provide useful tools to meet the needs of clinical relevancy in drug development and personalized medicine [65,66]. We established a co-culture system of pancreatic TSs and PSCs in microchannel chips where cancer cells could form spheroids within a collagen matrix. Quantitative analysis on confocal images of immunofluorescence staining in the present study should not compromise the validity of results, not only because of the quantitative potential of this technique [67], but also the well-controlled experimental conditions for imaging and analysis. Pooled sample analysis such as Western blotting is considered inappropriate for studying phenotypic heterogeneity in cell populations. Our model accommodated cancer cell–cancer cell (contact and juxtacrine), cancer cell–PSC (paracrine), and cell–ECM interactions and can be modified to study the influence of various tumor microenvironment factors. Using this system, we were able to investigate differential modes of 3D migration of cancer cells into ECM and concurrent remodeling of the collagen and fibronectin matrix in clinically relevant conditions. Overall, our pancreatic TS-PSC co-culture model represents a novel tool not only for studying phenotypic heterogeneity and plasticity in cell invasive behavior, but also for screening the anti-invasive activity of anticancer agents.

## 4. Materials and Methods

### 4.1. Cell Culture

The human pancreatic cancer cell lines PANC-1 and BxPC-3 were purchased from the American Type Culture Collection (Manassas, VA, USA). The human pancreatic stellate cell (PSC) line was obtained from ScienCell (HPaSteC, #3830, Carlsbad, CA, USA). PANC-1 cells and PSCs were maintained in high-glucose DMEM (Hyclone, Logan, UT, USA) and BxPC-3 cells were maintained in RPMI-1640 medium (Gibco, Grand Island, NY, USA); both media were supplemented with 100 μg/mL streptomycin, 100 units/mL penicillin, 250 ng/mL amphotericin B, and 10% fetal bovine serum (FBS; Welgene, Daegu, Korea). Cell culture was maintained in a humidified atmosphere (5% CO_2_/95% air) at 37 °C.

### 4.2. Fabrication of the Polydimethylsiloxane (PDMS) Microchannel Chip

Microchannel chips were designed to contain seven units in a single plate. Each unit contained three channels for cell loading and four medium channels. The channel width and depth was 700 µm and 190 µm, respectively. Microchannel chips were prepared using PDMS (Sylgard 184, Dow Chemical, Midland, MI, USA) according to the previously reported protocol [22,23]. An SU-8 patterned master was custom-made (AMED, Seoul, Korea) using photolithography, which was then applied to conventional soft lithography to produce PDMS replicas. In brief, the PDMS base and cross-linking agent were mixed thoroughly at a ratio of 10:1 (*w/w*), poured onto the master mold, and cured for 3 h at 60 °C. Upon removal from the master, inlet and outlet ports were formed using a 16 G needle and 4 mm disposable biopsy punch. The open sides of the PDMS replicas were secured to a glass coverslip with oxygen plasma (CUTE; Femto Science, Seoul, Korea). The formed microchannels were then coated with poly-dopamine solution (2 mg/mL) to promote type I collagen adhesion onto the channel surface, as previously reported [68]. The microchannel chips were then ready to be used after drying overnight in a 60 °C oven.

### 4.3. Three-Dimensional Spheroid Culture in the Microchannel Chip

PANC-1 and BxPC-3 cells were suspended at 7 × 10^5^ cells/mL and 1 × 10^6^ cells/mL, respectively, in 2 mg/mL collagen I solution (rat tail tendon type I collagen, Corning, NY, USA). Cells were loaded into each designated channel in 3 μL of a cell–hydrogel mixture at 2.1 × 10^3^ cells/channel for PANC-1 cells and at 3 × 10^3^ cells/channel for BxPC-3 cells. The ratio of cancer cells to PSCs was 1:1.4. After gelation in a cell culture incubator for 30 min, the microchannels were filled with culture medium and returned to the incubator for further culture for 5–7 days with daily medium changes. Cellular migration tracking was carried out over 10 h using a live-cell imaging system (Lionheart FX, BioTek, Winooski, VT, USA).

### 4.4. Immunofluorescence Staining and Image Acquisition

Immunostaining of TSs and PSCs was performed using 4% paraformaldehyde for 20 min and 0.5% Triton X-100 for another 30 min in the microchannels after fixation and permeabilization, respectively. After blocking non-specific binding using 10% normal goat serum overnight, the TSs and PSCs were incubated with primary antibodies overnight at 4 °C, followed by exposure to secondary antibodies (Alexa Fluor 594, A27016 or Alexa Fluor 488, A11034, 1:1000 for both; Thermo Fisher Scientific, Waltham, MA, USA). The following primary antibodies were used: Ki-67 (1:100, sc-15402, Santa Cruz, Dallas, TX, USA), pFAK (1:100, 44624G, Thermo Fisher Scientific), integrin β1 (1:200, ab24693, Abcam, Cambridge, UK), E-cadherin (1:300, 3195S, Cell Signaling Technology, Danvers, MD, USA), vimentin (1:600, ab92547, Abcam), transforming growth factor beta-1 (TGF-β1; 1:200, ab92486, Abcam), connective tissue growth factor (CTGF; 1:200, ab6992, Abcam), tissue inhibitor of metalloproteinases (TIMP-1; 1:100, 102D1, Thermo Fisher Scientific), type I collagen (1:200, ab34710, Abcam), fibronectin (1:200, ab2413, Abcam), MMP-2 (1:200, ab92536, Abcam), MMP-13 (1:100, MAB511-100, R&D Systems, Minneapolis, MN, USA), and collagen type I, cleavage site (Col1-¾C; 1:100, #0217-050, immunoGlobe, Würzburg, Germany). Staining of membrane type 1-matrix metalloproteinase (MT1-MMP; 1:100, ab78738, Abcam) was performed without permeabilization. F-actin was stained with rhodamine phalloidin (1:1000, R415, Invitrogen, Carlsbad, CA, USA) and DAPI was used for nuclear counterstaining (1:1000, D9564, Sigma-Aldrich, St. Louis, MO, USA). Image acquisition was achieved using confocal microscopy (LSM 800 W/Airyscan, Carl Zeiss, Oberkochen, Germany) and the fluorescence intensity was determined using ZEN software (Carl Zeiss). Confocal microscopy 3D image stacks were projected onto a single image, creating two-dimensional (2D) maximum-pixel-intensity z-projection images. The images were from three out of four accessible fields, which covered 80% of the effective area in each channel. For quantitative comparison, data were normalized to DAPI intensity.

### 4.5. Image Analysis

ImageJ software (National Institutes of Health, Bethesda, MD, USA) was used to analyze the confocal microscopy images. The apparent diameter (D) of TSs was calculated using the equation D = 2 × (area/π)^1/2^, assuming a spherical shape of TSs, in which the area was measured using ImageJ. Cells up to 20 μm in diameter were counted as single cells based on the individual cell size of 10 to 20 μm, and cell aggregates larger than 20 μm were considered TSs. The projected images were analyzed in ImageJ to quantify the deformed area. Structural organization of matrix fibers was analyzed for orientation and thickness. A coherency value closer to 1 indicates better matrix fiber alignment and deposition ability.

### 4.6. Statistical Analysis

All data are expressed as the mean ± standard error of three or more independent measurements. Student’s t-Test was used to test the statistical significance using Microsoft Excel 2010 (Redmond, WA, USA). *P* values < 0.05 were considered statistically significant.

## Supplementary Materials

The following are available online at https://www.mdpi.com/2072-6694/12/5/1305/s1, Figure S1: Expression of α-SMA and migration of PSCs when co-cultured with or without tumor spheroids (TSs). No significant difference in the level of α-SMA (**A**) and number of cells migrated out of collagen channels (**B**) under TSs co-culture conditions. Cells were grown for 5 days in collagen-supported microchannel chips. Scale bar: 100 µm, Figure S2: Expression of MT1-MMP in PANC-1 cells migrating with invadopodia. Cells were cultured in a 3D collagen matrix for 5 days and immunostaining was performed without a permeabilization step. Scale bar: 20 µm, Figure S3: Formation of tumor spheroids (TSs) of MIA PaCa-2 and Capan-1 cells and expression of EMT markers when co-cultured with or without PSCs. (**A**) Morphology of membrane protrusion shown by actin-based podia (white arrow). (**B**) Expression of EMT marker proteins E-cadherin and vimentin. Cells were grown for 5 days in collagen-supported microchannel chips and stained with F-actin, E-cadherin, vimentin and DAPI. Scale bar: 50 µm. * *p* < 0.05, Figure S4: (**A**) Expression of fibronectin with or without serum in a cell-free collagen matrix. (**B**) Expression of fibronectin in regions close to the TSs of PANC-1 and BxPC-3 cells. Immunostaining was performed at day 5 of culture. Scale bar: 50 µm (**A**, **B**).
