# Supplementary material for: Phenotypic Heterogeneity and Plasticity of Cancer Cell Migration in a Pancreatic Tumor Three-Dimensional Culture Model
Source: Cancers (Basel). 2020 May 21;12(5):1305. doi: 10.3390/cancers12051305 (PMC7281339; doi:10.3390/cancers12051305)
Supplement: Supplementary file 1 [file cancers-12-01305-s001.pdf]

Supplementary Materials

Phenotypic Heterogeneity and Plasticity of Cancer Cell Migration in a Pancreatic Tumor Three-Dimensional Culture Model

Seul-Ki Kim, So Dam Jang, Hyunho Kim, Seok Chung, Jong Kook Park and Hyo-Jeong Kuh

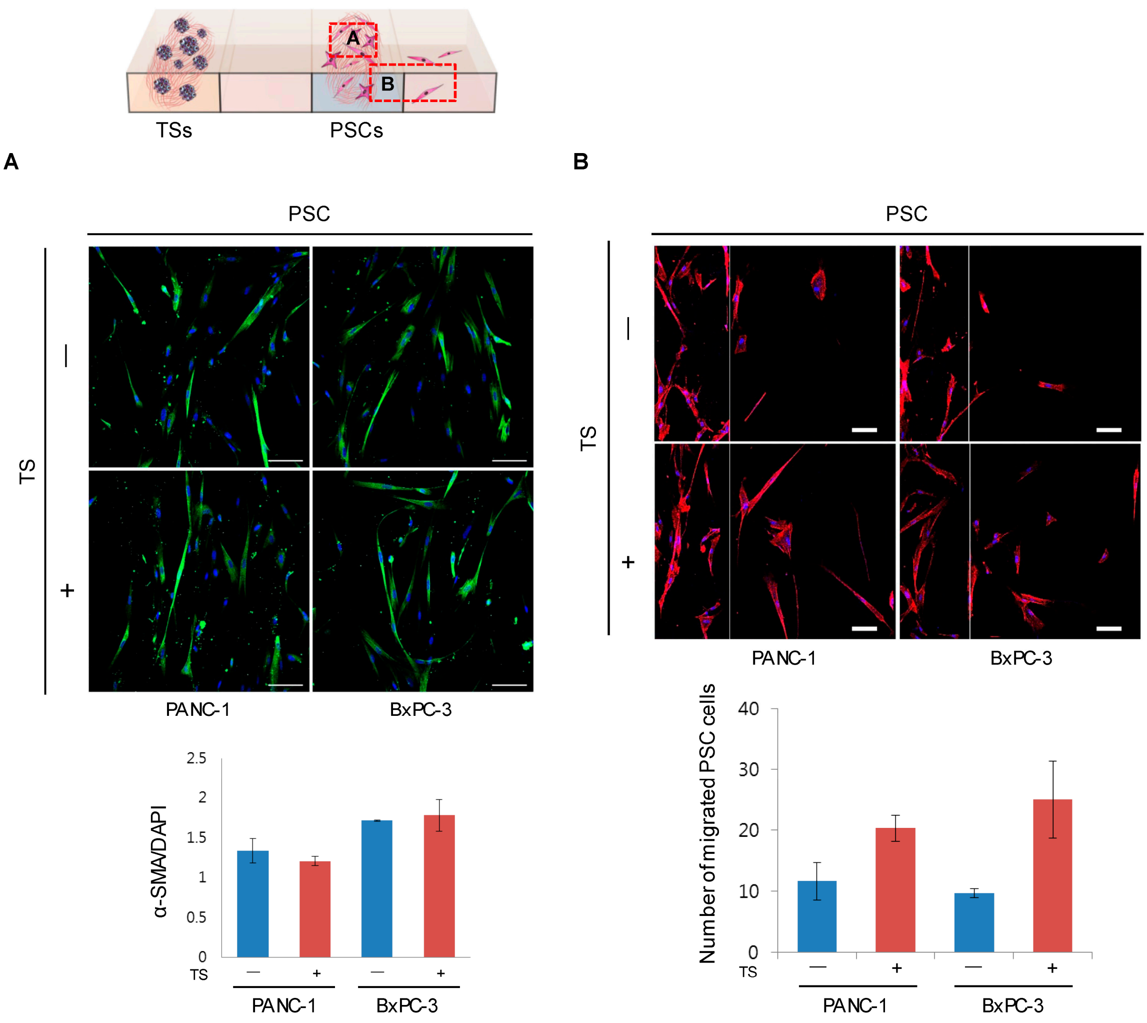

Figure S1. Expression of α-SMA and migration of PSCs when co-cultured with or without tumor spheroids (TSs). No significant difference in the level of α-SMA (**A**) and number of cells migrated out of collagen channels (**B**) under TSs co-culture conditions. Cells were grown for 5 days in collagen-supported microchannel chips. Scale bar: 100 μm.

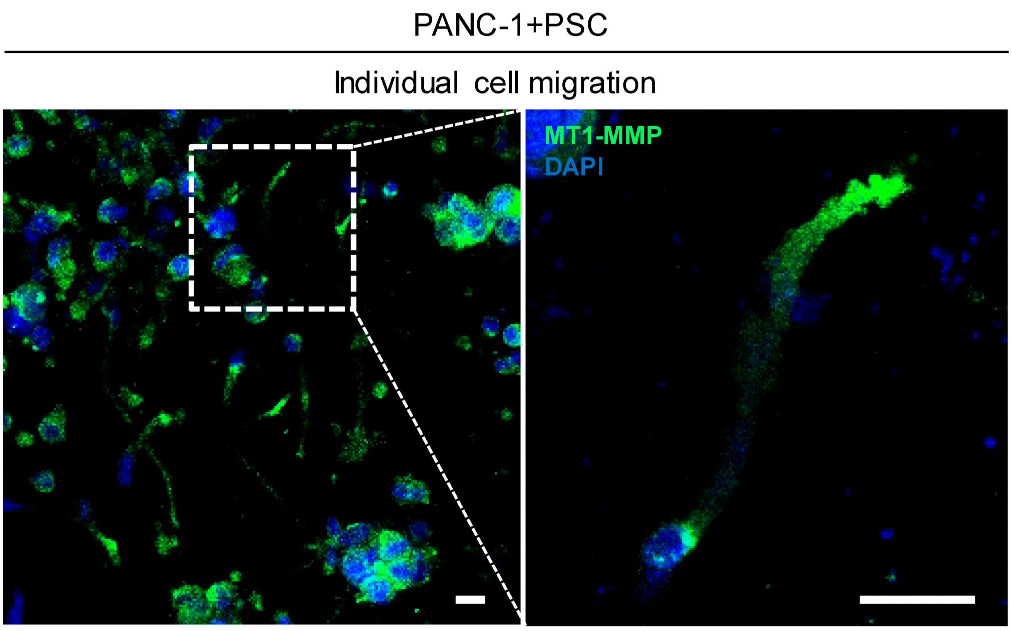

Figure S2. Expression of MT1-MMP in PANC-1 cells migrating with invadopodia. Cells were cultured in a 3D collagen matrix for 5 days and immunostaining was performed without a permeabilization step. Scale bar: 20 µm.

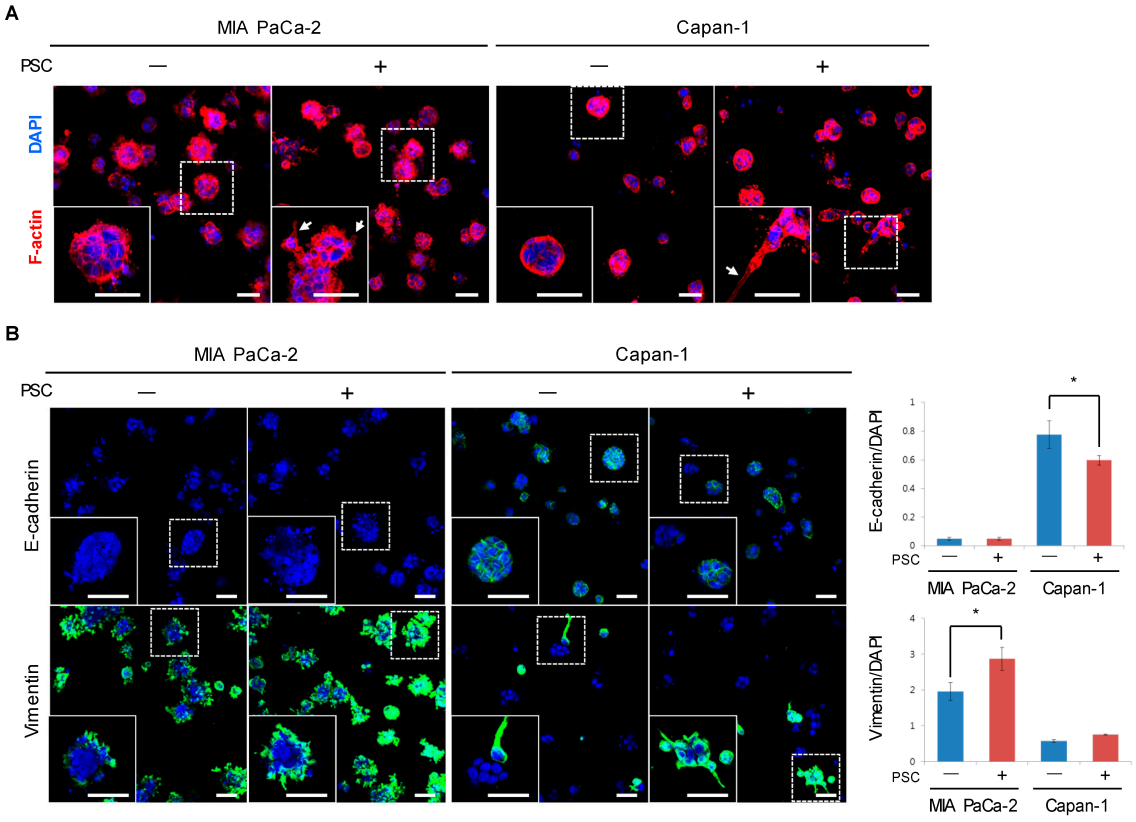

Figure S3. Formation of tumor spheroids (TSs) of MIA PaCa-2 and Capan-1 cells and expression of EMT markers when co-cultured with or without PSCs. (**A**) Morphology of membrane protrusion shown by actin-based podia (white arrow). (**B**) Expression of EMT marker proteins E-cadherin and

vimentin. Cells were grown for 5 days in collagen-supported microchannel chips and stained with F-actin, E-cadherin, vimentin and DAPI. Scale bar: 50 μm. * $p < 0.05$.

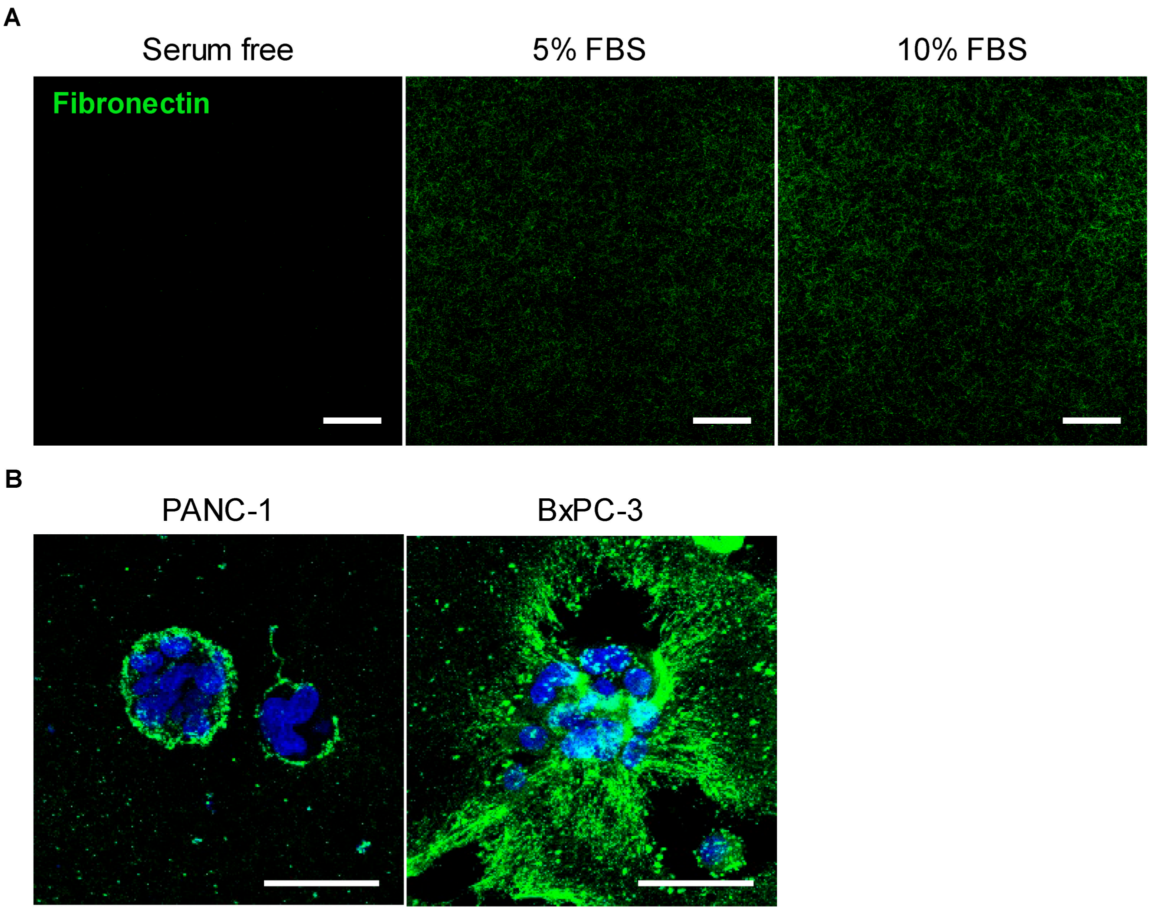

Figure S4. (**A**) Expression of fibronectin with or without serum in a cell-free collagen matrix. (**B**) Expression of fibronectin in regions close to the TSs of PANC-1 and BxPC-3 cells. Immunostaining was performed at day 5 of culture. Scale bar: 50 μm (**A**, **B**).

© 2020 by the authors. Licensee MDPI, Basel, Switzerland. This article is an open access article distributed under the terms and conditions of the Creative Commons Attribution (CC BY) license (http://creativecommons.org/licenses/by/4.0/).
